# Hyaluronic Acid-Based Gels and Biomaterial Systems for Oral Wound Healing: Design and Clinical Translation

**DOI:** 10.3390/gels12030262

**Published:** 2026-03-22

**Authors:** Vlad Constantin, Ionut Luchian, Dragos Ioan Virvescu, Mihaela Scurtu, Nicoleta Tofan, Dan Nicolae Bosinceanu, Elena Raluca Baciu, Carina Balcos, Monica Mihaela Scutariu, Dana Gabriela Budala

**Affiliations:** Grigore T. Popa University of Medicine and Pharmacy, 700115 Iasi, Romania

**Keywords:** hyaluronic acid, oral wound, oral mucosa, tissue regeneration, biomaterials

## Abstract

Hyaluronic acid (HA), a naturally occurring glycosaminoglycan and major component of the extracellular matrix, has attracted increasing interest as a therapeutic adjunct in oral wound management due to its biological activity and biocompatibility. The search for the current narrative review literature was performed in the PubMed/MEDLINE, Scopus, and Web of Science databases for studies published up to December 2025. Eligible studies included experimental investigations, clinical trials, and relevant reviews assessing HA applications in oral mucosal or gingival wounds. Available clinical evidence suggests potential benefits of HA in reducing postoperative discomfort, accelerating re-epithelialization, and improving soft tissue healing following periodontal and surgical procedures. However, substantial heterogeneity exists regarding molecular weight, formulation, concentration, and application protocols, which limits direct comparison between studies and precludes definitive conclusions. Further well-designed, standardized clinical trials are required to clarify optimal formulations and confirm long-term therapeutic benefits.

## 1. Introduction

Oral wound healing represents a critical process in dental medicine, directly influencing functional recovery, patient comfort, and long-term clinical outcomes. Oral wounds arise frequently as a consequence of surgical procedures, tooth extractions, periodontal therapy, implant placement, traumatic injuries, or inflammatory and ulcerative conditions of the oral mucosa [1,2]. Although oral tissues display a relatively high regenerative capacity compared to other body sites, impaired or delayed healing remains a common clinical challenge, often associated with pain, infection risk, compromised tissue integrity, and suboptimal esthetic or functional results [3]. Effective management of oral wounds is therefore essential for achieving predictable healing and improving overall oral health-related quality of life [4].

The oral cavity presents a uniquely complex environment for wound healing. Continuous exposure to saliva, a diverse and dynamic microbiota, mechanical stress generated by mastication and speech, and constant temperature and pH fluctuations can interfere with clot stability, epithelial migration, and tissue remodeling [5,6]. In addition, oral wounds are often subject to repeated trauma and contamination, which may prolong inflammation and increase susceptibility to secondary infection [7].

A cascade of cellular and molecular mechanisms is initiated in response to oral mucosa injury, facilitating tissue regeneration [8]. Wounds can penetrate deeper layers of skin and even nearby structures and systems such as muscles, tendons, blood vessels, nerves, and bone [9]. Surgical procedures, infections, trauma, and systemic diseases such as diabetes and autoimmune illnesses are among the numerous possible causes of these injuries to the oral cavity.

Hyaluronic acid (HA), a naturally occurring glycosaminoglycan and major component of the extracellular matrix, plays a fundamental role in tissue hydration, structural organization, and cellular signaling [6,7]. Its molecular characteristics, including high water-binding capacity, viscoelasticity, and receptor-mediated biological activity, make it a promising candidate for therapeutic applications in oral wound management. Importantly, HA exhibits size-dependent biological effects, with high- and low-molecular-weight fractions exerting distinct immunomodulatory and pro-regenerative functions [10,11].

Although hyaluronic acid-based materials are increasingly used in clinical dentistry, the available evidence remains heterogeneous, with variations in formulation, molecular weight, concentration, and application protocols. A critical synthesis of current biological and clinical data is therefore necessary to clarify therapeutic indications, limitations, and translational relevance.

The aim of this narrative review is to provide an overview of the biological mechanisms, material characteristics, and clinical performance of hyaluronic acid-based systems in oral wound healing, highlighting current evidence and future research directions.

## 2. Literature Overview and Selection Approach

Hyaluronic acid, a naturally occurring glycosaminoglycan widely distributed in connective tissues and a major component of the extracellular matrix, plays a fundamental role in tissue hydration, cell migration, angiogenesis, and inflammation control. Its biological functions are particularly relevant during the inflammatory and proliferative phases of wound repair [10]. Hyaluronic acid-based materials have therefore emerged as promising therapeutic agents for oral wound management, offering the potential to enhance tissue regeneration, reduce inflammatory burden, and promote faster and more organized healing [11].

### 2.1. Study Design

This work was conducted as a narrative review of the literature focusing on the effects of hyaluronic acid-based materials in the healing of oral wounds. The objective of this review was to provide an integrated overview of biological mechanisms, material-related characteristics, and clinical applications of hyaluronic acid-based systems in the oral environment.

Given the heterogeneity of available evidence and variability in study designs, a qualitative integrative approach was considered more appropriate than statistical pooling.

### 2.2. Literature Search Strategy

A structured literature search was performed using major electronic databases, including PubMed/MEDLINE, Scopus, and Web of Science. The search covered publications up to December 2025 to ensure inclusion of both foundational and contemporary evidence. These databases were selected for their comprehensive coverage of biomedical, dental, and materials science literature. Additional relevant studies were identified through manual screening of reference lists from key articles.

The search strategy combined controlled vocabulary and free-text terms related to hyaluronic acid and oral wound healing, including but not limited to hyaluronic acid, hyaluronan, oral wound healing, oral mucosal lesions, gingival healing, periodontal wounds, post-extraction healing, oral surgery, and oral biomaterials. Boolean operators (AND, OR) were used to optimize search sensitivity and specificity. Only studies published in English were considered.

### 2.3. Eligibility Criteria

Studies were included if they met the following criteria:

(i) original research articles, clinical trials, or relevant review papers;
(ii) evaluation of hyaluronic acid-based materials applied to oral wounds or oral mucosal healing;
(iii) assessment of biological effects, clinical outcomes, or mechanisms of action related to wound repair in the oral cavity.

Studies were excluded if they met any of the following criteria:

i. investigations not involving oral tissues or not specifically addressing oral wound healing;
ii. studies evaluating hyaluronic acid in non-dental medical contexts (e.g., dermatology, orthopedics, ophthalmology) without direct translational relevance to the oral environment;
iii. articles lacking sufficient methodological detail or clear outcome reporting;
iv. conference abstracts, editorials, letters to the editor, and non-peer-reviewed publications;
v. non-English language publications.

### 2.4. Data Extraction and Synthesis

Data were extracted qualitatively from eligible studies with attention to study design, type of hyaluronic acid formulation, molecular characteristics, mode of application, biological effects, and reported clinical outcomes. Comparative evaluation focused on identifying consistent biological effects, differences between formulations, and recurring methodological limitations across studies. Particular emphasis was placed on translational relevance and clinical applicability.

### 2.5. Quality Considerations and Limitations

Although no formal risk-of-bias assessment tool was applied, study quality was evaluated descriptively based on experimental design, sample size, clarity of outcome measures, and relevance to clinical practice. The inherent heterogeneity of available evidence and the predominance of in vitro and short-term clinical studies were recognized as key limitations of existing literature. Given the variability in outcome measures, follow-up duration, and study design, a narrative synthesis approach was employed rather than statistical pooling.

## 3. Biological Background of Oral Wound Healing

Oral wound healing is governed by a series of regulated biological phases and influenced by multiple local and systemic factors specific to the oral environment. Wound healing constitutes a highly complex biological process, characterized by coordinated cellular activities, precise gene regulation, and activation of intricate signaling pathways that collectively drive the sequential phases of tissue regeneration [10,11]. A detailed comprehension of these processes enables researchers to identify the principal molecular mediators that regulate tissue repair, including key signaling molecules, regulatory proteins, and components of the extracellular matrix.

### 3.1. Phases of Oral Wound Healing

The inflammatory phase is initiated immediately after tissue injury and is characterized by hemostasis, vascular permeability, and the recruitment of inflammatory cells, including neutrophils and macrophages [12]. These cells play a crucial role in debris clearance, microbial defense, and the release of cytokines and growth factors that orchestrate subsequent healing events. While a controlled inflammatory response is necessary for effective repair, excessive or prolonged inflammation may impair epithelialization and promote tissue breakdown, underscoring the importance of balanced immune regulation in oral wound healing [13,14].

The proliferative phase involves active cellular migration and proliferation, leading to the formation of granulation tissue. Fibroblasts synthesize extracellular matrix components, particularly collagen, while endothelial cells promote angiogenesis to restore local blood supply [15]. Concurrently, epithelial cells migrate across the wound surface to re-establish mucosal continuity. In the oral cavity, this phase is strongly influenced by mechanical stability and the biochemical composition of saliva, which can either facilitate or disrupt cellular activity at the wound site [16].

**Figure 1:**
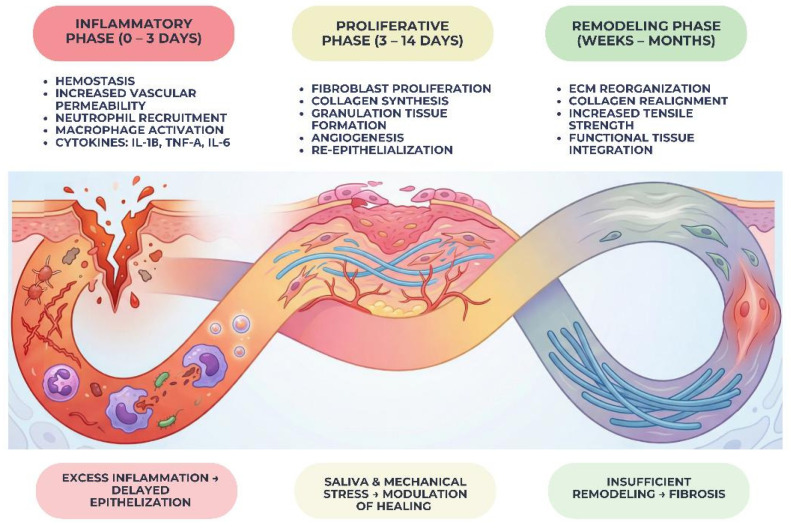
Mechanisms involved in oral wound healing. Schematic representation of the main phases of oral wound healing, including hemostasis, inflammation, proliferation, and remodeling. The upper part of the figure illustrates the biological events characteristic of each phase, such as platelet activation, inflammatory cell recruitment, fibroblast proliferation, angiogenesis, and extracellular matrix remodeling. The lower part highlights factors that may interfere with the normal progression of these phases, including microbial contamination, excessive inflammation, oxidative stress, and impaired tissue regeneration. In the remodeling stage, the elements illustrated in the lower section represent potential consequences of dysregulated healing, such as fibrosis, delayed tissue maturation, or compromised mucosal integrity. This conceptual scheme illustrates how biological and environmental factors can influence the balance between successful tissue regeneration and impaired wound healing.

The remodeling phase represents the final stage of wound healing and may extend over weeks or months. During this phase, newly formed extracellular matrix undergoes reorganization and maturation, with collagen fibers being realigned and replaced to enhance tissue strength and functionality [17]. In oral tissues, remodeling is critical for achieving long-term wound stability and functional integration, particularly in areas subjected to continuous mechanical loading. Inadequate remodeling may result in fibrotic tissue formation or compromised mucosal resilience [18]. To facilitate a structured understanding of the dynamic sequence of biological events involved in oral wound repair, Figure 1 schematically illustrates the three principal phases of oral wound healing—namely the inflammatory, proliferative, and remodeling stages—within the specific context of the oral environment. Figure 1 highlights the temporal progression of these phases and emphasizes the key cellular and molecular mechanisms that characterize each stage.

### 3.2. Factors Influencing Healing in the Oral Cavity

The unique combination of local and systemic variables that govern the healing of oral wounds sets them apart from wounds in other parts of the body. A complex and ever-changing microbial ecology, the mouth microbiota is one of the most important local variables. While commensal microbes help maintain dental homeostasis, problems with microbial balance or an overabundance of bacteria at wound sites can prolong inflammation, raise infection risks, and slow healing [19].

At least two functions of saliva are involved in the healing of oral wounds. Hydration is only one of its many beneficial effects; it also contains enzymes, antimicrobial peptides, and growth factors that aid in tissue regeneration and epithelial repair [20]. However, local wound care strategies may be hindered by continual salivary flow, which can potentially damage clot stability and limit the residence duration of topical therapeutic medicines.

Mechanical stress represents another critical factor influencing oral wound healing. Mastication, speech, and parafunctional habits subject oral tissues to repeated microtrauma, which can disrupt newly formed tissue and interfere with epithelial migration. This mechanical challenge is particularly relevant in post-surgical wounds and in areas adjacent to mobile mucosa, where wound stability is difficult to maintain [21,22].

**Figure 2:**
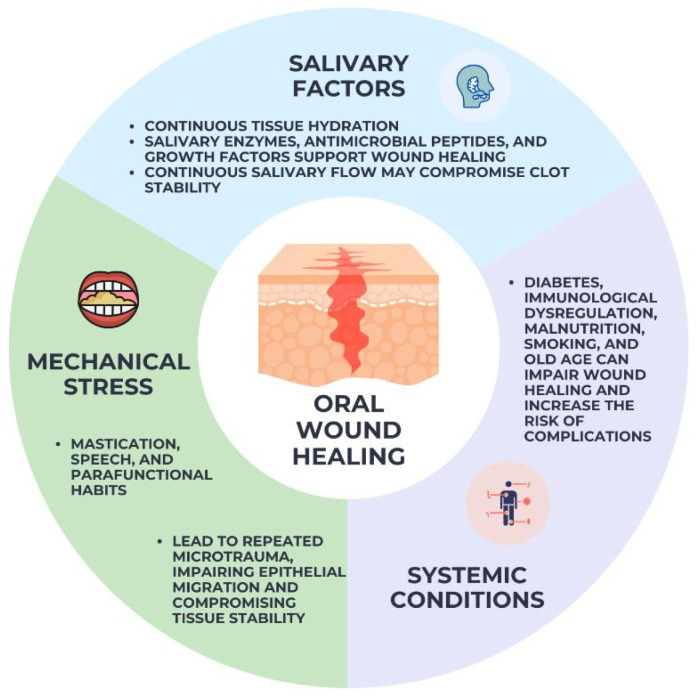
Factors influencing oral wound healing.

The oral cavity’s healing response is further modulated by systemic circumstances. Impaired wound healing and a higher risk of complications have been linked to metabolic illnesses, including diabetes mellitus, immunological dysregulation, malnutrition, smoking, and old age. Because of these systemic effects, inflammatory responses, vascular function, and cellular activity are all changed, which impacts the oral tissues’ ability to regenerate [23]. Optimizing therapeutic approaches and enhancing clinical outcomes in oral wound management requires an understanding of the combined impact of these parameters. Figure 2 illustrates the main local and systemic factors that influence oral wound healing. It highlights the dual role of saliva, the impact of mechanical stress from mastication and speech, and the contribution of systemic conditions such as metabolic disorders and aging. Together, these factors create a dynamic healing environment that can either support or impair tissue repair.

## 4. Hyaluronic Acid—Structure and Biological Functions

Hyaluronic acid is a naturally occurring, non-sulfated glycosaminoglycan widely distributed throughout connective tissues and a fundamental component of the extracellular matrix. In oral tissues, it plays a central role in maintaining tissue hydration, structural integrity, and cellular homeostasis [24]. Owing to its unique physicochemical properties and biological activity, hyaluronic acid has attracted increasing interest as a therapeutic agent in wound healing, particularly within the challenging environment of the oral cavity.

Chemically, hyaluronic acid consists of repeating disaccharide units composed of D-glucuronic acid and N-acetyl-D-glucosamine, linked by alternating β-1,3 and β-1,4 glycosidic bonds. This linear polymer lacks protein cores and sulfate groups, distinguishing it from other glycosaminoglycans and conferring high biocompatibility [25,26]. The molecular weight of hyaluronic acid can vary widely, ranging from low-molecular-weight fragments to high-molecular-weight polymers exceeding several million Daltons [27].

Molecular weight is a critical determinant of hyaluronic acid’s biological behavior. High-molecular-weight hyaluronic acid is generally associated with anti-inflammatory, anti-edematous, and protective effects, contributing to tissue homeostasis and barrier function. In contrast, low-molecular-weight hyaluronic acid fragments are more biologically active and can stimulate cell migration, angiogenesis, and immune cell recruitment [27,28,29].

Low-molecular-weight hyaluronic acid fragments generated during tissue injury or enzymatic degradation may act as endogenous danger-associated molecular patterns (DAMPs). These small fragments, particularly those below approximately 20 kDa, are capable of activating innate immune pathways through interaction with pattern-recognition receptors such as Toll-like receptor-4 (TLR-4), thereby promoting inflammatory signaling and recruitment of immune cells. In contrast, high-molecular-weight hyaluronic acid (generally exceeding 1000 kDa) contributes to tissue homeostasis by forming a highly hydrated extracellular matrix that acts as a protective physical barrier. This large polymer network stabilizes the tissue microenvironment, limits excessive inflammatory activation, and supports resolution of inflammation during the wound healing process.

**Figure 3:**
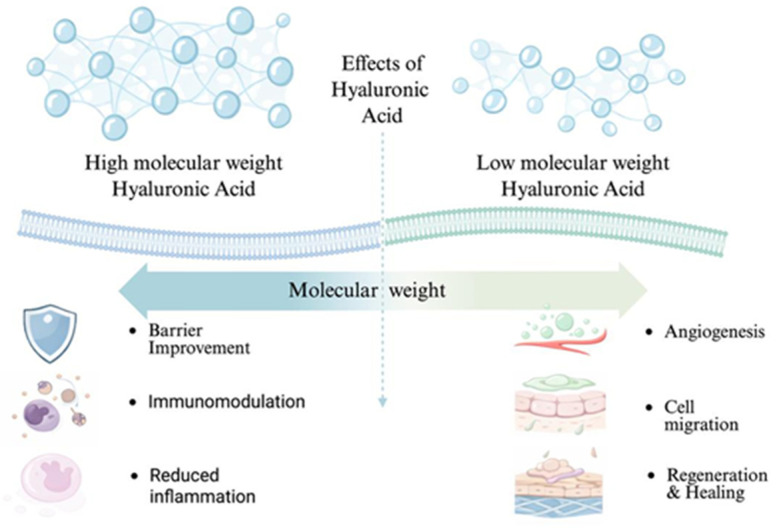
Hyaluronic acid molecular weight-dependent effects.

The size-dependent biological effects of hyaluronic acid in oral wound healing are schematically illustrated in Figure 3. The diagram highlights the distinct functional profiles of high- and low-molecular-weight hyaluronic acid and their respective contributions to inflammation control, barrier stabilization, angiogenesis, and tissue regeneration.

### 4.1. Physiological Role of Hyaluronic Acid in Tissues

Under physiological conditions, hyaluronic acid contributes to the viscoelastic properties of connective tissues and facilitates the organization of the extracellular matrix. Its strong hydrophilic nature enables the retention of large volumes of water, maintaining tissue hydration and creating a permissive environment for cell movement. In oral tissues, this function supports mucosal resilience and adaptability to mechanical stress [28,30].

Hyaluronic acid also plays a structural role by interacting with extracellular matrix proteins, cell surface receptors, and growth factors. Through these interactions, it regulates cellular adhesion, proliferation, and migration. During tissue injury, local concentrations of hyaluronic acid increase, reflecting its involvement in early wound healing responses [29,31]. The dynamic turnover of hyaluronic acid within tissues allows rapid adaptation to inflammatory and reparative stimuli.

During the inflammatory phase, hyaluronic acid contributes to the regulation of immune cell activity and cytokine signaling, helping to limit excessive inflammatory responses that may otherwise impair healing [32].

In the proliferative phase, hyaluronic acid facilitates fibroblast migration and proliferation, supports extracellular matrix deposition, and enhances endothelial cell activity, thereby promoting neovascularization [33]. These effects are essential for granulation tissue formation and for restoring vascular supply to the healing site. Additionally, hyaluronic acid supports epithelial cell migration and differentiation, accelerating re-epithelialization and re-establishment of mucosal continuity [34].

Beyond its direct cellular effects, hyaluronic acid contributes to the creation of a provisional extracellular matrix that stabilizes the wound environment and protects newly formed tissue from mechanical and microbial challenges. Its biodegradability and natural presence in tissues further support its suitability for therapeutic use, as degradation products are physiologically metabolized without eliciting adverse reactions [35,36].

**Figure 4:**
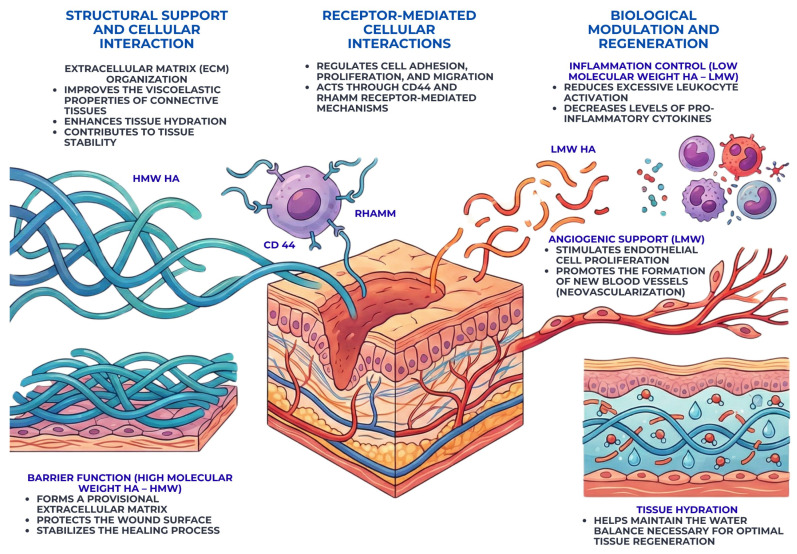
Schematic representation of the structural, receptor-mediated, and biological roles of hyaluronic acid (HA) in oral wound healing. High-molecular-weight HA (HMW-HA) contributes to extracellular matrix stability, viscoelastic properties, and barrier function, supporting tissue integrity and protection. The central panel illustrates HA interactions with CD44 and RHAMM receptors, regulating cell adhesion, migration, and tissue remodeling. Low-molecular-weight HA (LMW-HA) is associated with inflammatory signaling and angiogenic processes involved in tissue repair. The lower-right section highlights HA-mediated tissue hydration, essential for maintaining extracellular matrix homeostasis and optimal healing conditions.

Figure 4 summarizes the multifaceted roles of hyaluronic acid in oral wound repair, emphasizing its structural support, receptor-mediated cellular interactions, and molecular weight-dependent biological effects.

### 4.2. Mechanisms of Action of Hyaluronic Acid in Oral Wound Healing

The therapeutic relevance of hyaluronic acid in oral wound healing arises from its ability to interact with multiple biological pathways involved in inflammation, cellular migration, angiogenesis, and extracellular matrix remodeling. Rather than acting as a passive scaffold, hyaluronic acid functions as a dynamic regulator of the wound microenvironment, influencing both cellular behavior and tissue-level responses [35,36,37,38].

One of the primary mechanisms through which hyaluronic acid contributes to wound healing is the modulation of the inflammatory response. High-molecular-weight hyaluronic acid is known to exert anti-inflammatory effects by limiting excessive leukocyte activation and reducing the expression of pro-inflammatory cytokines [39]. This regulatory role is particularly important in the oral cavity, where prolonged inflammation may result from continuous microbial exposure and mechanical irritation. By promoting a controlled inflammatory phase, hyaluronic acid supports the transition toward tissue regeneration rather than chronic inflammation [40,41].

**Figure 5:**
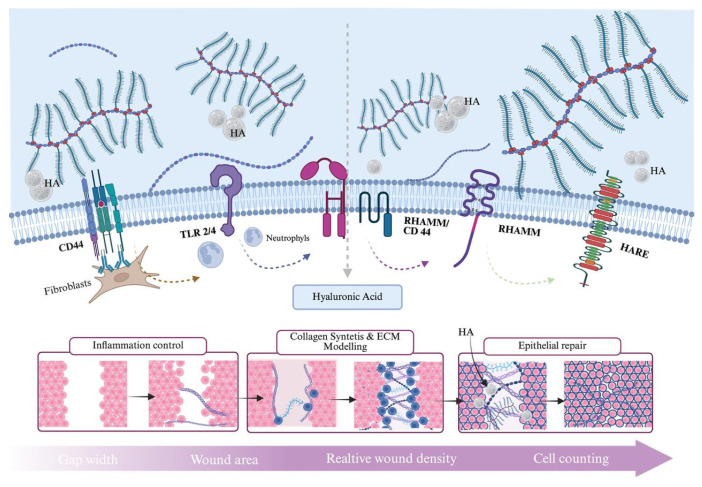
Receptor-Mediated Mechanisms of Hyaluronic Acid in Oral Wound Healing.

Hyaluronic acid also plays a critical role in facilitating cell migration and proliferation during the proliferative phase of wound healing. Its hydrophilic nature and capacity to form a hydrated extracellular matrix create a permissive environment for fibroblast and epithelial cell movement [42]. Interactions between hyaluronic acid and cell surface receptors, such as CD44 and RHAMM, regulate cytoskeletal organization and signal transduction pathways associated with cell motility and proliferation [43]. These interactions are essential for granulation tissue formation and re-epithelialization of oral wounds. The receptor-mediated mechanisms through which hyaluronic acid regulates inflammation, cell migration, angiogenesis, and extracellular matrix remodeling are schematically summarized in Figure 5.

Angiogenesis represents another key mechanism influenced by hyaluronic acid. Through both direct and indirect signaling pathways, hyaluronic acid supports endothelial cell proliferation and the formation of new blood vessels within the wound bed [44,45]. Low-molecular-weight hyaluronic acid fragments, in particular, have been associated with pro-angiogenic activity, contributing to the restoration of vascular supply necessary for oxygen and nutrient delivery [44]. This angiogenic support is especially relevant in oral tissues, where rapid vascular regeneration facilitates efficient healing [46].

In addition to its cellular effects, hyaluronic acid contributes to extracellular matrix organization and tissue remodeling. During the later stages of wound healing, hyaluronic acid regulates collagen deposition and matrix reorganization, promoting the formation of functionally aligned connective tissue [47]. Its gradual degradation and replacement by more mature matrix components allow a smooth transition from provisional tissue to stable oral mucosa. This process is essential for achieving long-term wound stability and resistance to mechanical stress [48,49].

Finally, hyaluronic acid exerts a protective role at the wound surface by forming a viscoelastic barrier that helps maintain hydration and shields the wound from mechanical trauma and microbial invasion [50]. This barrier function is particularly advantageous in the oral cavity, where constant movement and salivary flow may disrupt early healing. By stabilizing the wound microenvironment, hyaluronic acid enhances the overall efficiency and predictability of oral wound repair [51,52,53].

## 5. Hyaluronic Acid-Based Materials Used in Oral Wound Management

Due to its adaptability in terms of both physicochemical properties and its positive biological profile, hyaluronic acid has found its way into numerous formulations aimed at oral wound treatment [54]. Bioresorbable membranes, injectable preparations, mouth rinses, sprays, and topical gels are the most prevalent forms of HA in clinical dentistry [55]. A more modern approach to increasing mechanical stability and the amount of time HA spends in the mouth involves the use of cross-linked and composite HA devices.

Hyaluronic acid-based biomaterials used in oral wound management can be formulated in several material configurations depending on the intended clinical function and required mechanical performance. The most common forms include topical gels, cross-linked hydrogel systems, injectable formulations, membranes, and three-dimensional scaffolds [56]. Each configuration provides distinct physicochemical characteristics that influence retention time, mechanical stability, and biological interaction with oral tissues.

Topical HA gels are primarily designed to provide hydration, temporary barrier protection, and modulation of inflammatory responses at the wound surface. In contrast, chemically cross-linked HA systems are engineered to increase structural persistence and resistance to enzymatic degradation within the oral environment [57,58]. Membranes and scaffold-based constructs represent more advanced biomaterial systems that combine structural support with biological activity, enabling guided tissue regeneration and enhanced cellular migration [59].

The clinical performance of these materials depends not only on the intrinsic biological properties of hyaluronic acid but also on formulation parameters such as molecular weight distribution, cross-linking density, polymer concentration, and rheological behavior [55]. Consequently, understanding the material design principles of HA-based systems is essential for optimizing their therapeutic performance in oral wound healing.

Different hyaluronic acid-based biomaterials used in oral wound management are obtained through distinct preparation and fabrication strategies that directly influence their structural organization and clinical performance. Conventional HA gels are typically prepared by dissolving purified hyaluronic acid in aqueous media followed by controlled neutralization and homogenization to obtain viscoelastic formulations suitable for topical application [60]. Cross-linked HA hydrogels are generated through chemical or physical cross-linking reactions involving agents such as 1,4-butanediol diglycidyl ether (BDDE), carbodiimide systems, or divinyl sulfone, which create three-dimensional polymer networks with improved mechanical stability and resistance to enzymatic degradation [61].

More advanced HA-based materials, including membranes and porous scaffolds, are fabricated using techniques such as freeze-drying, electrospinning, or composite blending with other natural polymers such as collagen or chitosan. These fabrication strategies influence key properties including porosity, degradation kinetics, mechanical strength, and interaction with surrounding tissues [62]. Gel formulations offer ease of application and good adaptation to mucosal surfaces, but their clinical residence time may be limited by salivary dilution and mechanical stress.

Conversely, cross-linked hydrogels and scaffold-based constructs provide improved structural persistence and controlled degradation, although their production may involve more complex processing steps. Consequently, the selection of an appropriate HA-based material form requires balancing processability, mechanical stability, and biological performance within the oral wound environment.

### 5.1. Topical HA Gels and Solutions

Topical hyaluronic acid (HA) gels represent the most widely utilized formulation in oral wound management due to their ease of application, high biocompatibility, and capacity to conform to irregular mucosal surfaces. Unlike solid biomaterial systems, gels offer immediate adaptability to complex anatomical contours and allow uniform coverage of exposed connective tissue, surgical flaps, or ulcerative lesions. Most commercially available formulations are based on low- to medium-molecular-weight HA, typically dispersed in aqueous or hydrogel matrices at concentrations ranging between 0.1% and 0.8% [56,57,58].

The primary therapeutic mechanism of topical HA gels relies on the formation of a hydrated viscoelastic layer over the wound surface. This hydrated matrix performs multiple simultaneous functions: it maintains local moisture, reduces friction-induced trauma during mastication and speech, supports epithelial cell migration, and creates a temporary barrier against microbial and mechanical insult [57]. In addition, the high water-binding capacity of HA contributes to maintaining osmotic balance and stabilizing the provisional extracellular matrix during early healing stages.

From a formulation standpoint, gel viscosity and rheological behavior are critical determinants of clinical performance. Ideally, HA gels should exhibit shear-thinning properties—facilitating easy application under mechanical stress while preserving structural integrity at rest [55,57]. Insufficient viscosity leads to rapid dispersion and dilution in saliva, whereas excessively viscous formulations may compromise spreadability and patient comfort. Achieving an optimal balance between mucoadhesion and handling properties remains a central design challenge [58].

Nečas et al. reported that exposure of hyaluronic acid to alkaline environments leads to pronounced and largely irreversible degradation, characterized by a significant reduction in molecular weight and a marked decline in viscoelastic properties. Such chemical instability becomes especially problematic in applications that rely on prolonged structural integrity and sustained mechanical performance, including intra-articular formulations and dermal filler materials [59].

Hyaluronic acid (HA) solutions display non-Newtonian flow characteristics, specifically pseudoplastic (shear-thinning) behavior. This rheological profile results from the progressive disruption of intermolecular hydrogen bonding and hydrophobic associations as shear stress increases. Under flow conditions, HA polymer chains undergo deformation and orient along the direction of movement, leading to a measurable reduction in viscosity at higher shear rates [63].

Importantly, HA solutions are generally considered non-thixotropic, meaning that their structural organization and viscosity are restored once the applied shear is reduced. The transient disassembly of the polymer network is therefore reversible, with the system returning to its initial conformational state after mechanical stress ceases. This reversible shear-responsive behavior is fundamental to HA’s functionality and largely explains its widespread use in biomedical and biotechnological applications, where injectability, spreadability, and structural recovery are essential performance parameters [64].

Salivary ionic composition can significantly influence the rheological behavior and structural stability of hyaluronic acid gels applied in the oral cavity. Electrolytes present in saliva, including sodium, calcium, and phosphate ions, may alter electrostatic interactions between polymer chains, affecting chain entanglement and the viscoelastic properties of the hydrogel network [63]. Increased ionic strength may partially screen repulsive charges along the HA foundation, promoting chain contraction and reducing the effective viscosity of the system. For topical dental gels to resist salivary dilution and mechanical displacement, appropriate viscoelastic parameters are required [64].

In general, HA gels designed for intraoral applications should exhibit sufficient viscosity and a dominant elastic component, reflected by a storage modulus (G′) higher than the loss modulus (G″), ensuring structural integrity and improved resistance to salivary washout. Optimization of these rheological properties is therefore critical for maintaining gel retention on mucosal surfaces and ensuring sustained biological activity at the wound site [65].

Clinical studies report beneficial effects of adjunctive HA gel use in periodontal surgery, tooth extraction, peri-implant procedures, and management of mucosal ulcers, including reduced postoperative discomfort, decreased edema, and accelerated re-epithelialization [66,67]. However, interpretation of these findings is complicated by substantial heterogeneity in formulation parameters. Variations in molecular weight, cross-linking status, concentration, dosing frequency, and treatment duration limit direct comparability between studies [68,69].

### 5.2. Cross-Linked HA Systems

To overcome rapid degradation and limited mechanical persistence, chemically cross-linked HA systems have been introduced [70]. Cross-linking enhances structural stability, prolongs bioavailability, and may improve controlled release of active molecules within the wound environment [71]. These formulations demonstrate improved resistance to enzymatic breakdown by hyaluronidases and offer extended anti-inflammatory and regenerative effects.

Cross-linked hyaluronic acid systems used in oral wound management are, in most clinical scenarios, reticulated gel formulations specifically engineered to improve structural stability and prolong intraoral residence time. Chemical cross-linking introduces covalent bonds between HA polymer chains, generating a three-dimensional network that increases mechanical integrity and resistance to enzymatic degradation [72].

In the oral cavity, where salivary dilution, mechanical stress, and hyaluronidase activity rapidly reduce the persistence of non-cross-linked formulations, cross-linking becomes a critical design strategy [73]. By limiting polymer chain mobility, reticulated HA gels demonstrate slower degradation kinetics, improved viscoelastic stability, and enhanced retention on mucosal surfaces. This prolonged bioavailability may allow sustained interaction with cell surface receptors such as CD44 and RHAMM, potentially amplifying biological effects during the inflammatory and proliferative phases of healing [74], as can be seen in Figure 5.

In addition to CD44 and RHAMM, hyaluronic acid can interact with multiple signaling pathways, including Toll-like receptors (particularly TLR2 and TLR4) and other receptor-mediated mechanisms involved in inflammatory and regenerative responses. These pathways contribute to the complex biological activity of hyaluronic acid, particularly in modulating immune responses and tissue repair. However, CD44- and RHAMM-dependent signaling remains the most extensively characterized and functionally relevant mechanisms in the context of oral wound healing.

An additional parameter influencing the biological performance of cross-linked hyaluronic acid systems is the degree of modification (MoD), which reflects the proportion of HA functional groups involved in cross-linking reactions [74]. While cross-linking agents such as BDDE or divinyl sulfone improve mechanical stability and resistance to enzymatic degradation, excessive modification may negatively affect biological functionality [75].

High cross-linking density can restrict polymer chain mobility and potentially mask receptor-interacting regions along the hyaluronic acid polysaccharide chain, including domains involved in CD44-mediated cellular interactions [76]. Consequently, optimal hydrogel design requires a balanced degree of modification that preserves receptor accessibility while maintaining sufficient structural stability and resistance to degradation.

To better contextualize the structural variability of cross-linked hyaluronic acid (HA) gel systems, Table 1 summarizes the principal cross-linking strategies employed to obtain HA-based hydrogels, including their underlying mechanisms, advantages, limitations, and translational relevance in oral applications. Importantly, the design of cross-linked hyaluronic acid systems must balance mechanical persistence with biological visibility. While increased cross-linking density enhances structural stability and resistance to enzymatic degradation, excessive modification may reduce receptor accessibility and limit interactions with cell surface receptors such as CD44. Achieving an optimal balance between mechanical integrity and biological functionality is therefore essential for maintaining the regenerative potential of HA-based biomaterials.

The comparative overview highlights how different chemical and physical approaches directly influence mechanical stability, degradation kinetics, and clinical performance.

Overall, the data presented in Table 1 emphasize that the choice of cross-linking strategy critically determines the balance between mechanical stability and biological performance of hyaluronic acid-based systems. Covalent cross-linking approaches, such as BDDE and DVS, provide superior structural integrity and prolonged degradation, making them suitable for applications requiring extended retention; however, they may be associated with increased rigidity and potential cytotoxicity if not carefully controlled. In contrast, carbodiimide and enzymatic cross-linking methods offer improved biocompatibility and more favorable biological interactions, although with moderate mechanical strength. Physical and self-assembly-based systems exhibit excellent safety and simplicity but are limited by reduced stability and susceptibility to salivary dilution. Therefore, the optimal design of HA-based materials for oral applications requires a tailored approach, balancing durability, bioactivity, and clinical practicality depending on the therapeutic indication.

### 5.3. HA-Based Membranes and Scaffolds

In more advanced regenerative contexts, HA has been incorporated into bioresorbable membranes and three-dimensional scaffolds used in periodontal and surgical applications [84]. These systems may serve both as physical barriers and as bioactive matrices that promote cell migration, angiogenesis, and extracellular matrix organization [85].

Unlike surface-applied gels, these constructs provide structural support in addition to biological modulation, making them particularly relevant in periodontal and surgical contexts where spatial stability and guided tissue regeneration are required [86].

Three-dimensional HA scaffolds extend this concept by providing a porous architecture that supports cell adhesion, migration, and proliferation. These scaffolds may be fabricated through freeze-drying, electrospinning, or advanced bio fabrication techniques, generating interconnected pore networks that enable nutrient diffusion and vascular ingrowth. In oral regenerative medicine, such systems have been investigated for periodontal defects, peri-implant soft tissue reconstruction, and alveolar bone regeneration [86,87].

HA-based membranes are typically engineered through cross-linking or composite fabrication techniques to enhance mechanical strength and control degradation kinetics.

Composite biomaterials combining HA with collagen, chitosan, or other natural polymers have demonstrated improved mechanical strength and enhanced cellular compatibility [88]. These hybrid systems attempt to balance hydration capacity with structural integrity, particularly in sites exposed to mechanical stress [88].

### 5.4. Clinical Applications and Limitations

Clinically, hyaluronic acid (HA)-based materials have been employed in a broad range of oral conditions, including post-extraction socket management, periodontal flap surgery, peri-implant soft tissue procedures, mucosal ulcerations, and the treatment of inflammatory or traumatic lesions [89,90,91]. In these contexts, HA is generally used as an adjunctive therapy aimed at enhancing physiological healing rather than replacing conventional surgical protocols. Table 2 summarizes the main clinical indications in which hyaluronic acid-based materials have been evaluated for oral wound management.

Overall, the clinical data summarized in Table 2 indicate that hyaluronic acid-based formulations provide consistent benefits in oral wound healing, particularly in reducing postoperative pain, inflammation, and edema, while promoting faster epithelialization and improved soft tissue stability. These effects are most evident in short-term applications, such as post-extraction sockets, periodontal surgery, and oral mucosal lesions. In peri-implant contexts, hyaluronic acid appears to support early mucosal healing and tissue integration, although long-term outcomes remain insufficiently documented. Despite these positive findings, the available evidence is limited by small sample sizes, heterogeneity in study design and HA formulations, and relatively short follow-up periods. Therefore, while current data support the clinical usefulness of hyaluronic acid in oral wound management, further standardized and long-term studies are required to confirm its efficacy and optimize its therapeutic indications.

Despite these encouraging findings, interpretation of the clinical evidence remains challenging. Study designs vary considerably, ranging from small-scale randomized trials to observational or pilot studies, often with limited sample sizes and short follow-up durations. Outcome measures are frequently heterogeneous, including subjective pain scores, clinical healing indices, photographic assessments, or surrogate biological markers, which limit direct comparability across investigations. Moreover, many studies lack standardized control protocols or uniform surgical techniques, further complicating evidence synthesis, as shown in Table 3.

### 5.5. Design Principles and Translational Challenges of HA-Based Gels in Oral Wound Healing

Hyaluronic acid-based gels represent the most widely applied formulation in oral wound management due to their ease of application, biocompatibility, and ability to conform to irregular mucosal surfaces. However, the therapeutic performance of HA gels is highly dependent on rational formulation design rather than solely on the intrinsic biological properties of hyaluronic acid [96].

From a materials science perspective, the effectiveness of HA gels is governed by several critical parameters, including molecular weight distribution, concentration, degree of cross-linking, viscoelastic behavior, and degradation kinetics. These factors directly influence gel consistency, retention time in the oral cavity, diffusion properties, and biological interaction with the wound microenvironment [102].

One of the primary design challenges in oral applications is ensuring sufficient residence time under dynamic conditions characterized by salivary flow, mechanical stress, and enzymatic degradation. Non-cross-linked HA gels, although biologically active, are prone to rapid dilution and clearance. Increasing polymer concentration may improve viscosity and mucoadhesion but can simultaneously alter spreadability and patient comfort. Conversely, cross-linking strategies enhance structural stability and resistance to hyaluronidase-mediated degradation, yet excessive cross-link density may reduce molecular mobility and potentially limit receptor-mediated biological activity [87,91,94].

Rheological optimization is therefore central to gel design. Ideal HA gels for oral wound healing should exhibit shear-thinning behavior, allowing easy application while maintaining structural integrity at rest. Additionally, balanced water retention capacity is required to maintain hydration without promoting excessive swelling or mechanical instability.

Despite promising short-term clinical outcomes, translational challenges remain. Variability in commercial formulations, inconsistent reporting of molecular weight specifications, and lack of standardized concentration ranges complicate cross-study comparisons. Furthermore, few studies systematically correlate physicochemical properties of HA gels with clinical endpoints, limiting mechanistic understanding of formulation–performance relationships.

## 6. Discussion

Although hyaluronic acid-based materials demonstrate promising biological and clinical potential in oral wound management, several areas require further investigation to optimize their therapeutic performance and translational relevance. Future research should focus on refining formulation strategies, enhancing bioavailability, and improving standardization of clinical protocols.

One key direction involves the development of advanced cross-linked and chemically modified HA systems designed to prolong residence time within the oral cavity. Given the challenges posed by salivary dilution and enzymatic degradation, improved stabilization strategies—such as controlled cross-linking density or hybrid polymer systems—may enhance sustained bioactivity and reduce the need for repeated application.

Nanostructured HA-based delivery platforms represent another emerging area of interest. Incorporation of HA into nanoparticle systems, hydrogels, or injectable matrices may enable controlled release of bioactive agents, including antimicrobial compounds, anti-inflammatory drugs, or growth factors. Such multifunctional systems could provide combined structural support and targeted molecular modulation within the wound microenvironment.

Further research is also needed to clarify the size-dependent biological effects of HA in oral tissues. Standardized classification of molecular weight ranges and systematic evaluation of dose–response relationships would improve comparability across studies and facilitate evidence-based clinical recommendations.

From a translational perspective, well-designed multicenter randomized controlled trials with adequate sample sizes and long-term follow-up are essential. Harmonization of outcome measures—such as validated healing indices, quantitative inflammatory markers, and patient-reported outcome measures—would strengthen the clinical evidence base.

Finally, integration of HA-based systems into regenerative protocols combining platelet concentrates, stem cell approaches, or bioactive scaffolds may represent a promising frontier in oral tissue engineering. A mechanistic understanding of HA-mediated signaling pathways, including CD44 and RHAMM interactions, will further support rational biomaterial design and personalized therapeutic strategies.

## 7. Conclusions

The growing interest in hyaluronic acid-based materials reflects a broader shift toward biologically guided strategies in oral tissue management. Rather than serving merely as passive wound dressings, these systems interact dynamically with the healing microenvironment, influencing cellular behavior and extracellular matrix organization.

Clinical benefits are supported in early healing phases; however, formulation heterogeneity remains a critical barrier to standardization.

Future progress must be design-driven, correlating physicochemical parameters with reproducible clinical outcomes. Only through rigorous material optimization can HA gels achieve predictable translational reliability in oral regenerative practice.

## Figures and Tables

**Table 1:** Comparative Overview of Hyaluronic Acid Cross-Linking Strategies for Oral and Periodontal Applications.

Cross-Linking Strategy	Common Cross-Linkers/Method	Mechanism	Advantages	Limitations	Typical Oral Application Relevance
Chemical Covalent Cross-Linking (Ether-based) [77]	BDDE (1,4-butanediol diglycidyl ether)	Epoxide reaction with HA hydroxyl groups forming stable ether bonds	High mechanical stability; prolonged degradation; strong viscoelasticity	Residual toxicity risk; excessive rigidity if over-crosslinked	Extended retention gels for post-surgical wounds
Carbodiimide Cross-Linking [78]	EDC/NHS coupling	Amide bond formation between HA carboxyl and amine groups	No permanent foreign linker; tunable cross-link density	More complex synthesis; moderate elasticity	Biocompatible gels for mucosal healing
Divinyl Sulfone (DVS) Cross-Linking [77]	Divinyl sulfone	Michael-type addition to hydroxyl groups	Strong, durable network; high structural integrity	Potential cytotoxicity; limited dental clinical use	Experimental high-strength hydrogel systems
Physical Ionic Cross-Linking [79]	Multivalent ions (e.g., Ca^2+^)	Electrostatic interactions between charged HA chains	No chemical residues; reversible system	Lower stability; faster enzymatic degradation	Short-term protective oral gels
Hydrogen Bonding/Self-Assembly [80]	pH/temperature-induced structuring	Intermolecular hydrogen bonding and chain entanglement	Simple; highly biocompatible	Weak persistence; salivary dilution sensitivity	Mild topical oral formulations
Photo-Cross-Linking [81,82]	Methacrylated HA + light activation	Radical polymerization forming a covalent network	Precise spatial control; tunable stiffness	Requires photo initiators; limited intraoral practicality	Advanced regenerative scaffolds
Enzymatic Cross-Linking [83]	Transglutaminase-mediated systems	Enzyme-driven covalent bond formation	Mild reaction conditions; high biological compatibility	Cost; limited commercial scalability	Bioengineered wound healing systems

**Table 2:** Clinical Evidence Summary: Hyaluronic Acid in Oral Wound Healing.

Clinical Indication	Type of Study	Reported Outcomes	Follow-Up Duration	Main Limitations
Post-extraction socket [92,93]	Randomized controlled trials/observational studies	Reduced pain; decreased edema; faster epithelial closure	Short-term (7–30 days)	Small sample sizes; heterogeneous protocols
Periodontal flap surgery [94,95]	Controlled clinical trials	Improved healing index; reduced inflammation; enhanced soft tissue stability	2–8 weeks	Variability in HA concentration and surgical techniques
Peri-implant soft tissue management [96,97,98,99]	Prospective clinical studies	Improved early mucosal healing; better tissue integration	Up to 3 months	Limited long-term outcome data
Oral mucosal ulcers (aphthous/inflammatory lesions) [100,101]	Clinical trials/pilot studies	Reduced lesion size; decreased symptom severity; shortened healing time	1–4 weeks	Short follow-up; subjective outcome measures
Post-surgical inflammatory lesions [102,103]	Observational studies	Reduced postoperative discomfort; improved tissue appearance	Short-term	Lack of standardized control groups

**Table 3:** Comparative Overview of Hyaluronic Acid-Based Formulations in Oral Wound Management.

Formulation Type	Molecular Characteristics	Main Clinical Indications	Advantages	Limitations
Topical HA Gel (Non-Crosslinked) [52,104]	Low-to-medium molecular weight (0.1–0.8%)	Post-extraction wounds; periodontal surgery; mucosal lesions	Easy application; hydration; anti-inflammatory effect; supports re-epithelialization	Short residence time; rapid enzymatic degradation; salivary dilution
Cross-Linked HA Gel [56,105]	Chemically stabilized HA; prolonged degradation time	Post-surgical wounds; regenerative procedures	Increased stability; prolonged bioactivity; improved tissue persistence	Higher cost; limited long-term clinical evidence
HA Injectable Formulations [67]	High-molecular weight; viscoelastic properties	Periodontal defects; soft tissue augmentation	Localized effect; volumetric support; enhanced tissue integration	Technique-sensitive; variable clinical protocols
HA-Based Membranes [85,86]	Cross-linked or composite (HA + collagen/chitosan)	Guided tissue regeneration; surgical wounds	Barrier function; structural support; bioactive matrix	Handling sensitivity; variability in degradation rate
HA Composite Scaffolds [87]	HA combined with natural or synthetic polymers	Advanced regenerative applications	Improved mechanical strength; controlled release potential	Limited clinical validation; largely experimental

## Data Availability

No new data were created or analyzed in this study. Data sharing is not applicable to this article.

## Abbreviations

The following abbreviations are used in this manuscript:

HA	Hyaluronic Acid
CD44	Cluster of Differentiation 44
RHAMM	Receptor for Hyaluronan-Mediated Motility
G′	Storage modulus (elastic modulus)
G″	Loss modulus (viscous modulus)
BDDE	1,4-butanediol diglycidyl ether
